# Detection of resistance mutations and CD4 slopes in individuals experiencing sustained virological failure

**DOI:** 10.7448/IAS.17.4.19737

**Published:** 2014-11-02

**Authors:** Anna Schultze, Roger Paredes, Caroline Sabin, Andrew N Phillips, Deenan Pillay, Ole Kirk, Jens D Lundgren, Anton Pozniak, Mark Nelson, Alessandro Cozzi-Lepri, on behalf of Eurosida in Eurocoord

**Affiliations:** 1 Department of Infection and Population Health, University College London, London, UK; 2 Hospital Universitari Germans Trias i Pujol, Universitat Autònoma de Barcelona, Fundacions irsiCaixa i Lluita contra la SIDA, Badalona, Spain; 3 CHIP, Department of Infectious Diseases, Rigshospitalet, University of Copenhagen, Copenhagen, Denmark; 4 HIV and Sexual Health – Kobler Clinic, Chelsea and Westminster Hospital, London, UK; 5 CHIP, Department of Infectious Diseases, Rigshospitalet, University of Copenhagen, Copenhagen, Denmark

## Abstract

**Introduction:**

Several resistance mutations have been shown to affect viral fitness, and the presence of certain mutations might result in clinical benefit for patients kept on a virologically failing regimen due to an exhaustion of drug options. We sought to quantify the effect of resistance mutations on CD4 slopes in patients undergoing episodes of viral failure.

**Materials and Methods:**

Patients from the EuroSIDA and UK CHIC cohorts undergoing at least one episode of virological failure (>3 consecutive RNA measurements >500 on ART) with at least three CD4 measurements and a resistance test during the episode were included. Mutations were identified using the IAS-US (2013) list, and were presumed to be present from detection until the end of an episode. Multivariable linear mixed models with a random intercept and slope adjusted for age, baseline CD4 count, hepatitis C, drug type, RNA (log-scale), risk group and subtype were used to estimate CD4 slopes. Individual mutations with a population prevalence of >10% were tested for their effect on the CD4 slope.

**Results:**

A total of 2731 patients experiencing a median of 1 (range 1–4) episodes were included in this analysis. The prevalence of any resistance per episode was 88.4%; NNRTI resistance was most common (78.5%). Overall, CD4 counts declined by 17.1 (−19.7; −14.5) cells per year; this decline was less marked with partial viral suppression (current HIV RNA more than 1.5 log below the setpoint; p=0.01). In multivariable models adjusting for viral load, CD4 decline was slower during episodes with detected resistance compared to episodes without detected resistance (21.0 cells/year less, 95% CI 11.75–30.31, p<0.001). Among those with more than one resistance mutation, there was only weak evidence that class-specific mutations had any effect on the CD4 slope (Table 1). The effects of individual mutations (incl. M184V) were explored, but none were significantly associated with the CD4 slope; for these comparisons, a Bonferroni-corrected p-value level was 0.003.

**Conclusions:**

In our study population, detected resistance was associated with slightly less steep CD4 declines. This may be due to a biological effect of resistance on CD4 slopes, or other unmeasured factors such as poor adherence among individuals without resistance. Among individuals with detected drug resistance, we found no evidence suggesting that the presence of individual mutations was associated with beneficial CD4 slope changes.

**Table 1:** The effect of class-specific mutations among individuals with>1 detected resistance mutation

Comparison	Difference in CD4 Slope (95% CI)	p
NRTI vs no NRTI	12.63 (1.94–23.32)	0.02
NNRTI vs no NNRTI	−5.77 (−11.99–0.45)	0.07
PI vs no PI	0.73 (−4.98–6.43)	0.75

